# Ambient Air Pollution and Stillbirths Risk in Sydney, Australia

**DOI:** 10.3390/toxics9090209

**Published:** 2021-08-31

**Authors:** Bin Jalaludin, Farhad Salimi, Mahsan Sadeghi, Laura Collie, Geoffrey Morgan

**Affiliations:** 1 Ingham Institute for Applied Medical Research, University of New South Wales, Sydney 2170, Australia; 2 Centre for Air Pollution, Energy and Health Research (CAR), Glebe 2037, Australia; mahsan.sadeghi@unsw.edu.au (M.S.); geoffrey.morgan@sydney.edu.au (G.M.); 3 School of Public Health and Preventive Medicine, Monash University, Melbourne 3004, Australia; farhad.salimi@monash.edu; 4 School of Built Environment, University of New South Wales, Sydney 2033, Australia; 5 New South Wales Ministry of Health, Sydney 2010, Australia; laura.collie@health.nsw.gov.au; 6 Sydney School of Public Health, Faculty of Medicine and Health, University of Sydney, Sydney 2006, Australia

**Keywords:** ambient air pollution, particulate matter, ozone, stillbirth

## Abstract

We aimed to determine the associations between ambient air pollution, specifically particulate matter less than or equal to 10 microns and 2.5 microns (PM_10_ and PM_2.5_ respectively) and ozone (O_3_), and stillbirths. We analysed all singleton births between 20–42 weeks gestation in metropolitan Sydney, Australia, from 1997 to 2012. We implemented logistic regression to assess the associations between air pollutants and stillbirth for each trimester and for the entire pregnancy. Over the study period, there were 967,694 live births and 4287 stillbirths. Mean levels of PM_10_, PM_2.5_ and O_3_ for the entire pregnancy were 17.9 µg/m^3^, 7.1 µg/m^3^ and 3.2 ppb, respectively. Adjusted odds ratios were generally greater than unity for associations between PM and stillbirths, but none were statistically significant. There were no significant associations between O_3_ and stillbirths. There was potential effect modification of the PM_10_ and O_3_ association by maternal age. We did not find consistent evidence of associations between PM and O_3_ and stillbirths in Sydney, Australia. More high quality birth cohort studies are required to clarify associations between air pollution and stillbirths.

## 1. Introduction

Stillbirth is a devastating pregnancy outcome. Globally, there were around 2.6 million stillbirths in 2015, with a global stillbirth rate of 18.4 stillbirths per 1000 total births with the greatest burden occurring in low- and middle-income countries, particularly in Sub-Saharan African and Southern Asia [1]. The stillbirth rates range between 25–30 per 1000 total births for South Asia and Sub-Saharan Africa to less than 5 per 1000 total births for the economically developed regions of the world [1].

Australia has a much lower stillbirth rate than the high risk regions with a stillbirth rate of 7.1 per 1000 total births, a rate that has remained relatively stable over the past two decades [2]. Stillbirths represent a considerable cost to the nation and community in psychological, physical, social and financial terms. In Australia, the value of lost social welfare from stillbirth was estimated at AU$18 billion (2017/18 dollars) in the 2015/16 financial year [3].

In high income countries, the major risk factors for stillbirths—such as advanced maternal age, obesity and smoking—account for 30% of all stillbirths [4]. Other risk factors include first pregnancy, and maternal diabetes and hypertension [5]. Importantly, many of these risk factors are modifiable or preventable. Therefore, investment in reducing stillbirths and improving support services could present cost-effective ways of minimising the economic impact, especially on already stressed health systems.

Several environmental factors have also now been implicated in the increased risks for stillbirths. In a review of meta-analyses of environmental exposures and pregnancy outcomes, Nieuwenhuijsen et al. [6] reported increased risks of stillbirths for environmental tobacco smoke, household air pollution from the use of solid fuels for cooking and heating, and disinfectant by-products in water.

Ambient air pollution is now considered to be the biggest environmental health risk globally and has adverse impacts on foetal growth and pregnancy outcomes [7]. However, there are few published studies and meta-analyses on the effects of ambient air pollution on stillbirths. In their 2013 review of meta-analysis, Nieuwenhuijsen et al. [6] did not find any meta-analysis of air pollution and stillbirths. However, in the most recently published meta-analysis of air pollution and stillbirths, Zhang et al. [8] reviewed 12 studies of long-term exposure to air pollutants and found increased risks for particulate matter less than or equal to less than 2.5 microns in diameter (PM_2.5_) exposure in the third trimester and the entire pregnancy (7 studies), carbon monoxide (CO, 6 studies) exposure in the third trimester (6 studies), and ozone (O_3_) in the first trimester (6 studies), and stillbirths. There were no associations between particulate matter less than or equal to less than 10 microns in diameter (PM_10_, 6 studies), nitrogen dioxide (NO_2_, 6 studies), sulphur dioxide (SO_2_, 6 studies), and stillbirth risks.

An Australian perspective on this issue is important. While Australian cities generally have low air pollution compared to similar economically developed economy cities, they do experience intermittent exposure to high concentrations of fine particles through bushfire smoke, which are projected to become more frequent and severe in the future (State of the Climate 2020; accessed on 17 August 2021 at https://www.csiro.au/en/Showcase/state-of-the-climate). If the association between ambient air pollution and stillbirth were to be confirmed, this would have significant public health implications for Australia. We therefore aimed to determine the associations between ambient air pollution, specifically PM_2.5_, PM_10_ and O_3_, with stillbirths in an Australian birth cohort.

## 2. Materials and Methods

The study area was metropolitan Sydney, New South Wales (NSW), Australia. We obtained data for all births in metropolitan Sydney (12,367 km^2^) for 1997 to 2012 from the NSW Ministry of Health’s Perinatal Data Collection (PDC). The PDC consists of information for all live births and stillbirths (death prior to birth) of at least 20 weeks gestation or at least 400 g. Information is collected on maternal antenatal factors, labour and delivery details as well as newborn details. We obtained information on infant’s gender, Indigenous status, whether the mother was born overseas, parity, antenatal care attendance, maternal smoking during pregnancy, gestational diabetes, maternal diabetes and maternal hypertension.

We used the date of the last menstrual period to estimate gestational age (conception two weeks following the last menstrual period). We included all births between 20 and 42 gestational weeks in our study. We excluded all non-singleton births.

We assessed neighbourhood area level socioeconomic status using the Index of Relative Socioeconomic Disadvantage (IRSD) from the Australian Bureau of Statistics [9]. IRSD is measured using a composite of several variables (e.g., income, education, and unemployment) within a neighbourhood, defined as a Census Collection District (CCD, spatial unit with an average 220 dwellings in urban area). Lower scores of IRSD indicate greater neighbourhood disadvantage. We calculated the quintiles of the neighbourhood IRSD score across NSW and assigned each individual in the study region to one of the IRSD quintiles.

### 2.1. Air Pollution Exposures

We obtained daily air pollution data for the study region (daily 1-h maximum O_3_ measured in parts per billion (ppb), daily average PM_10_ and PM_2.5,_ both measured in µg/m^3^) from the New South Wales Office of Environment and Heritage. O_3_ and PM data were available from eight fixed site monitors for the entire study period. Daily average concentration was only calculated when at least 75% of hourly data were available. If missing daily values for a site were less than 5% of days, the missing values were replaced with the mean value of previous and the following day for that site. Missing values on other days were replaced by the mean of the remaining sites multiplied by a weighting factor. We calculated the weighting factor by dividing the seasonal mean of the missing site by the corresponding mean from the other sites. Daily city wide mean value of the air pollutant concentration from all available monitoring sites was averaged and used to estimate pregnancy exposures (trimester specific exposures and for the entire pregnancy).

### 2.2. Statistical Analysis

We implemented logistic regression to assess the associations between exposure to air pollutants and odds of stillbirth. Separate analyses were conducted for each trimester and for the entire pregnancy. All models were adjusted for infant gender, maternal smoking during pregnancy (never smoker vs. ever smoker), Indigenous status (yes vs. no), first pregnancy (yes vs. no), antenatal care attendance ≤12 weeks gestation (yes vs. no), gestational diabetes (yes vs. no), maternal diabetes (yes vs. no), maternal hypertension (yes vs. no), mother’s country of birth (Australia vs. other), mother’s age (<35 years vs. ≥35 years), and neighbourhood level socioeconomic disadvantage (in quintiles). We also tested for effect modification by including maternal age and area level socioeconomic disadvantage (in quintiles) as interaction terms. We performed stratified analyses if the interaction terms were significant. A *p*-value of <0.05 denoted statistical significance.

We present odds ratios (ORs) and associated ninety-five percent confidence intervals (95%CIs) for 2 µg/m^3^ increase of PM_2.5_ concentration, 4 µg/m^3^ increase of PM_10_ concentration and 1 ppb increase in O_3_ concentration.

## 3. Results

Over the 16-year study period, after implementing the exclusion criteria, there were 967,694 live births and 4287 stillbirths (4.4/1000 births). Socio-demographic and clinical characteristics are presented in Table 1. Women who had a stillbirth were more likely to have diabetes and hypertension, be primiparous, more disadvantaged, and less than 35 years of age. 

The mean and median ambient PM and ozone concentrations were similar across all three trimesters and for the entire pregnancy (Table 2).

The adjusted odds ratios were generally greater than unity for associations between PM and stillbirths (Table 3). However, none of these associations were statistically significant. There were no significant associations between ozone and stillbirths (Table 3).

There was no effect modification by IRSD. We found evidence for effect modification by maternal age and PM_10_ in trimester 3 (for 4 ug/m^3^ increase: maternal age < 35 years: OR = 1.032, 95%CI = 0.987–1.077; maternal age ≥ 35 years: OR = 0.897, 95%CI = 0.817–0.977), for maternal age and ozone in trimester 2 (for 1 ppb increase: maternal age < 35 years: OR = 1.182, 95%CI = 1.064–1.313; maternal age ≥ 35 years: OR = 0.804, 95%CI = 0.670–0.965), and for maternal age and ozone in the entire pregnancy (for 1 ppb increase: maternal age < 35 years: OR = 1.134, 95%CI = 0.984–1.307; maternal age ≥ 35 years; OR = 0.685, 95%CI = 0.537–0.876).

## 4. Discussion

We did not find any associations between ambient air pollution and stillbirths in our 16-year study in Sydney, Australia. We captured all livebirths and stillbirths in Sydney and we also had individual level information on important risk factors for stillbirths on all women and particularly on cigarette smoking during pregnancy.

Our PM_10_ findings are consistent with those of Hwang et al. [10] who conducted a population-based case–control study in Taiwan with 9325 stillbirths and the control group of 93,250 births. They reported no associations between maternal exposure to PM_10_ (mean concentration 73 μg/m^3^) and increased risk of stillbirth in all births over 20 weeks gestation in Taiwan. Green et al. [11], in a retrospective study of about 14,000 stillbirths and just over three million livebirths in California showed increased, but non-significant, risk of stillbirths for PM_2.5_ exposure (mean concentration 15 μg/m^3^) during the entire pregnancy. However, De Franco et al. [12] in a population based study in the US (1848 stillbirths and 349,188 livebirths), reported a 42% increased rate of stillbirth associated with PM_2.5_ exposure (mean concentration 13 μg/m^3^), but only for exposure in the third trimester. Similarly, a hospital-based cohort study in Korea [13] found a 10 µg/m^3^ increase in PM_10_ during the third trimester was associated with an eight percent increase in stillbirth risk (67 stillbirths and 1447 livebirths; mean concentration 89 μg/m^3^). In a more recent population-based study (5377 stillbirths and 26,885 matched livebirths), Ebisu et al. [14] showed that PM_2.5_ (mean concentration 18 μg/m^3^) was associated with a significantly increased risk of stillbirths due to foetal growth complications, for example, small for gestation age. In the two large population-based studies [11,12], there are no consistent associations between PM and stillbirths.

The hospital-based study from Korea had a small size yet showed a significant association between PM_10_ and stillbirths. The reason for this could be that in their hospital-based birth cohort, Kim et al. [13] were able to collect detailed individual-level data relevant to stillbirths, for example, body mass index and smoking and alcohol consumption during pregnancy. Several reasons have been postulated for the inconsistent results among studies including differences in air pollution exposure assessment, definition of stillbirths, statistical methods and the availability of important covariates, leading to low to high heterogeneity in meta-analysis [8].

As with the studies on PM and stillbirths, the studies investigating O_3_ and stillbirths have also reported mixed results. Among the five cohort studies reporting associations between exposure to O_3_ and stillbirths, two failed to establish a link [10,15] where the ambient O_3_ concentrations were between 30 to 37 ppb and the number of stillbirths were 587 [15] and 9325 [10] and the other three studies found adverse effects of air pollution on stillbirths. A cohort study in US [16] (992 stillbirths and 222,383 livebirths) found a 39% increased risk of stillbirth associated with exposure to O_3_ during the entire pregnancy (mean O_3_ concentration 29 ppb) and a retrospective study in US (14,000 stillbirths and just over three million livebirths) found a one percent increase in stillbirth rate per 10 ppb increase in O_3_ exposure during the entire pregnancy period [11] (mean O_3_ concentration 48 ppb). In a recent study from the UK, Smith et al. [17] (578,382 livebirths and 3392 stillbirths; mean O_3_ concentration 31–33 ppb), showed an increased risk of stillbirths for exposure to O_3_ in the first and second trimesters (17% and 15%, respectively).

We found that maternal age, but not neighbourhood socioeconomic status (SES), modified the associations between PM_2.5_ and O_3_ and stillbirths. PM_10_ significantly decreased the risk of stillbirths in women ≥35 years of age whereas O_3_ significantly increased the risk of stillbirths in women <35 years of age but reduced the risk of stillbirths in women ≥35 years of age. Others have reported higher associations between PM_2.5_ [18,19] and O_3_ [11] and risks of stillbirths in women aged ≥35 years of age compared to women aged <35 years of age. As women may only be exposed for a short period in the third trimester prior to a stillbirth and because of the inconsistent results among the published studies, the findings of effect modification by maternal age should be cautiously interpreted.

There may be several reasons for our null findings. It should be noted that the concentrations of ambient PM in other studies were at least several orders of magnitude greater than in our study. The ambient PM concentrations in Sydney are low compared to other cities globally. For example, the mean concentration of PM_2.5_ over the entire pregnancy in this current study was only about 7 µg/m^3^ and for O_3_ it was 3 ppb. Although studies from North America on air pollution and mortality have shown adverse effects at low levels of PM_2.5_ (3-year annual average 6.7–8 µg/m^3^) [20], we did not find significant associations between PM_2.5_ (annual average 4.5 µg/m^3^) and mortality in Sydney [21]. We allocated the same city-wide level air pollution levels to all women in the study and we were unable to take into account the amount of time spent indoors, mobility and change of addresses during the pregnancy. Such exposure misclassification is generally non-differential and bias the effect estimates towards the null, and could explain our null results.

Our study has several strengths. It is a population level study and we used high quality administrative dataset that captures all births in metropolitan Sydney. Importantly, were able to control for important confounders, particularly maternal smoking during pregnancy, an important risk factor for stillbirths. None of the other studies reported here, except for one [13] were able to control for maternal smoking during pregnancy as this information, unlike in Australia, is generally not routinely collected and recorded in birth administrative datasets.

There are also some limitations to our study. Firstly, like most studies of air pollution and health, air pollution exposure was calculated at the ecological level rather than at the individual level. We also did not have information on some other important risk factors for stillbirths, such as body mass index, and hence were unable to control for them in our analysis. Our exposure estimates focussed on exposure over trimesters and over the entire pregnancy rather than the effects of acute intense air pollution episodes or shorter air pollution exposure windows. It is possible that acute intense air pollution episodes during pregnancy, for example, extreme smoke concentrations from bushfires, could impact on stillbirths and should be considered in future research.

## 5. Conclusions

We did not find associations between PM and O_3_ and stillbirths in Sydney, Australia, where ambient air pollution levels are relatively low compared to similar cities internationally. The literature on associations between air pollution and stillbirths is mixed. Multi-city studies, with large sample sizes, a range of ambient air pollution levels, controlling for critical confounders and risk factors, improved exposure assessment and using harmonised statistical methods can help in clarifying and building the evidence on the association between air pollution and stillbirths. As air pollution is a preventable potential risk factor for stillbirths, investment in mitigating air pollution would be prudent and could minimise the substantial social and economic impacts from stillbirths.

## Figures and Tables

**Table 1:** Characteristics of live births and stillbirths, Sydney, Australia, 1997–2012.

Characteristics	Live Births (*n* = 967,694) *n* (%)	Stillbirths (*n* = 4287) *n* (%)	Total (*n* = 971,981) *n* (%)	Chi-Square Test *p*-Value
Infant gender				0.022
Missing	536	18	554	
Female	468,760 (48.5)	1994 (46.7)	470,754 (48.5)	
Male	498,398 (51.5)	2275 (53.3)	500,673 (51.5)	
Maternal diabetes				<0.001
No	962,486 (99.5)	4245 (99.0)	966,731 (99.5)	
Yes	5208 (0.5)	42 (1.0)	5250 (0.5)	
Gestational diabetes				0.003
No	916,210 (94.7)	4103 (95.7)	920,313 (94.7)	
Yes	51,484 (5.3)	184 (4.3)	51,668 (5.3)	
Maternal hypertension				<0.001
No	959,854 (99.2)	4206 (98.1)	964,060 (99.2)	
Yes	7840 (0.8)	81 (1.9)	7921 (0.8)	
Maternal smoking				<0.001
No	863,171 (89.2)	3566 (83.2)	866,737 (89.2)	
Yes	104,523 (10.8)	721 (16.8)	105,244 (10.8)	
Indigenous status				<0.001
No	957,191 (98.9)	4209 (98.2)	961,400 (98.9)	
Yes	10,503 (1.1)	78 (1.8)	10,581 (1.1)	
Maternal country of birth				0.575
Australia	40,6279 (42.0)	1818 (42.4)	408,097 (42.0)	
Other	561,415 (58.0)	2469 (57.6)	563,884 (58.0)	
First pregnancy				<0.001
First	430,057 (44.4)	2070 (48.3)	432,127 (44.5)	
Multiparous	537,637 (55.6)	2217 (51.7)	539,854 (55.5)	
Antenatal care attendance				<0.001
No	563,197 (58.2)	2228 (52.0)	565,425 (58.2)	
Yes	404,497 (41.8)	2059 (48.0)	406,556 (41.8)	
Maternal age (years)				0.004
Missing	252	1	253	
<35	749,077 (77.4)	3206 (74.8)	752,283 (77.4)	
≥35	218,365 (22.6)	1080 (25.2)	219,445 (22.6)	
Index of Relative Socioeconomic Disadvantage				<0.001
Missing	2900	23	2923	
1st quintile (most disadvantaged)	204,908 (21.2)	1088 (25.5)	205,996 (21.3)	
2nd quintile	146,769 (15.2)	741 (17.4)	147,510 (15.2)	
3rd quintile	151,546 (15.7)	690 (16.2)	152,236 (15.7)	
4th quintile	154,592 (16.0)	669 (15.7)	155,261 (16.0)	
5th quintile (least disadvantaged)	306,979 (31.8)	1076 (25.2)	308,055 (31.8)	

**Table 2:** Mean, standard deviation (SD) and median ambient air pollution concentrations by trimesters and entire pregnancy, Sydney, Australia, 1997–2012.

Ambient Air Pollution Concentration	Mean (SD)	Median	Interquartile Range
1st trimester			
PM_10_ (µg/m^3^)	18.0 (4.1)	17.1	4.2
PM_2.5_ (µg/m^3^)	7.1 (1.8)	6.7	1.5
O_3_ (ppb)	3.2 (0.6)	3.2	0.9
2nd trimester			
PM_10_ (µg/m^3^)	17.8 (4.1)	16.9	4.1
PM_2.5_ (µg/m^3^)	7.1 (1.8)	6.7	1.5
O_3_ (ppb)	3.2 (0.6)	3.2	0.9
3rd trimester			
PM_10_ (µg/m^3^)	17.8 (4.2)	16.9	4.2
PM_2.5_ (µg/m^3^)	7.1 (1.8)	6.8	1.6
O_3_ (ppb)	3.2 (0.6)	3.2	0.9
Entire pregnancy			
PM_10_ (µg/m^3^)	17.9 (2.7)	17.4	3.6
PM_2.5_ (µg/m^3^)	7.1 (1.4)	6.8	1.4
O_3_ (ppb)	3.2 (0.3)	3.3	0.5

**Table 3:** Adjusted ^1^ odds ratios of stillbirth associated with ambient air pollution exposure, Sydney, Australia 1997–2012.

Ambient Air Pollution	Adjusted Odds Ratio for Stillbirth
PM_10_ (per 4 µg/m^3^ increase)	
Entire pregnancy	0.997 (0.952–1.004)
First trimester	1.001 (0.968–1.033)
Second trimester	1.000 (0.968–1.033)
Third trimester	1.000 (0.960–1.039)
PM_2.5_ (per 2 µg/m^3^ increase)	
Entire pregnancy	1.017 (0.972–1.064)
First trimester	1.024 (0.988–1.061)
Second trimester	1.002 (0.966–1.039)
Third trimester	0.990 (0.974–1.034)
O_3_ (per 1 ppb increase)	
Entire pregnancy	1.002 (0.887–1.133)
First trimester	0.987 (0.904–1.078)
Second trimester	1.074 (0.980–1.176)
Third trimester	0.902 (0.800–1.016)

^1^ Adjusted for infant’s gender, Indigenous status, gestational diabetes, maternal diabetes, maternal hypertension, smoking during pregnancy, whether first pregnancy, antenatal care after 12 weeks of pregnancy, maternal age, and area level socioeconomic status.

## Data Availability

The data presented in this study are held by the NSW Ministry of Health. The data are not publicly available due to privacy and confidentiality reasons.
